# Predictive validity of a brief antiretroviral adherence index: Retrospective cohort analysis under conditions of repetitive administration

**DOI:** 10.1186/1742-6405-5-20

**Published:** 2008-08-29

**Authors:** William C Mathews, Eva Barker, Erica Winter, Craig Ballard, Bradford Colwell, Susanne May

**Affiliations:** 1 UCSD Owen Clinic, UCSD Medical Center, Mail Code 8681, 200 W. Arbor Dr., San Diego, CA 92103, USA; 2 Division of Biostatistics and Bioinformatics, Department of Family and Preventive Medicine, University of California San Diego, Mail Code 0717, 9500 Gilman Dr., La Jolla, CA 92093, USA

## Abstract

**Background:**

Newer antiretroviral (ARV) agents have improved pharmacokinetics, potency, and tolerability and have enabled the design of regimens with improved virologic outcomes. Successful antiretroviral therapy is dependent on patient adherence. In previous research, we validated a subset of items from the ACTG adherence battery as prognostic of virologic suppression at 6 months and correlated with adherence estimates from the Medication Event Monitoring System (MEMS). The objective of the current study was to validate the longitudinal use of the Owen Clinic adherence index in analyses of time to initial virologic suppression and maintenance of suppression.

**Results:**

278 patients (naïve n = 168, experienced n = 110) met inclusion criteria. Median [range] time on the first regimen during the study period was 286 (30 – 1221) days. 217 patients (78%) achieved an undetectable plasma viral load (pVL) at median 63 days. 8.3% (18/217) of patients experienced viral rebound (pVL > 400) after initial suppression. Adherence scores varied from 0 – 25 (mean 1.06, median 0). The lowest detectable adherence score cut point using this instrument was ≥ 5 for both initial suppression and maintenance of suppression. In the final Cox model of time to first undetectable pVL, controlling for prior treatment experience and baseline viral load, the adjusted hazard ratio for time updated adherence score was 0.36_score ≥ 5 _(95% CI: 0.19–0.69) [reference: <5]. In the final generalized estimating equations (GEE) logistic regression model the adjusted odds ratio for time-updated adherence score was 0.17_score ≥ 5 _(0.05–0.66) [reference: <5].

**Conclusion:**

A brief, longitudinally administered self report adherence instrument predicted both initial virologic suppression and maintenance of suppression in patients using contemporary ARV regimens. The survey can be used for identification of sub-optimal adherence with subsequent appropriate intervention.

## Introduction

In previous research, we validated a subset of items from the ACTG adherence battery as prognostic of virologic suppression at 6 months and moderately correlated with adherence estimates from the Medication Event Monitoring System (MEMS) [1]. The objective of the current study was to validate the longitudinal use of the Owen Clinic adherence index in analyses of time to initial virologic suppression and maintenance of suppression.

## Results

Study eligibility criteria were met by 278 patients whose baseline characteristics are presented in Table 1. Participants were predominantly male (88%), middle aged (median 39 years), men having sex with men (MSM) (64%), white (47%), and antiretroviral therapy treatment naive (60%). The median absolute CD4+ lymphocyte count and log_10 _transformed HIV plasma viral load were 173 and 5.0, respectively. Index antiretroviral regimens were distributed as follows: ≥ 2 nucleoside reverse transcriptase inhibitors (NRTIs) + 1 boosted protease inhibitor (PI/r) 73%, ≥ 2 NRTIs + 1 non-nucleoside reverse transcriptase inhibitor (NNRTI) 23%, and other regimens 4%. Enfuvirtide was included as part of the index regimen in only two patients. Median [IQR] days on the index regimen was 286 [115–566] overall. According to prior antiretroviral experience, the median [IQR] days on therapy was 285 [116–566] for treatment naïve patients and 286 [93–562] for treatment experienced patients. 217 patients (78%) achieved an undetectable pVL at median 63 days. 8.3% (18/217) of patients experienced viral rebound (pVL > 400) after initial suppression. The median number of per-patient administrations of the adherence instrument was 4, varying from 1 to 27 administrations. Adherence scores varied from 0 – 25 (mean 1.06, median 0).

**Table 1:** Patient Characteristics at Study Entry (n = 278)

**Characteristic**	
Sex [n (%)]	
Female	33 (12)
Male	245 (88)
HIV Transmission Risk Factor [n (%)]	
MSM_1_, not IDU_2_	179 (64)
Heterosexual contact	52 (19)
IDU	23 (8)
Other/Unknown	24 (9)
Race/Ethnicity [n (%)]	
White	130 (47)
Black	30 (11)
Hispanic	87 (31)
Other/Unknown	31 (11)
Age (years)	
[mean (sd)]	39.5 (9.2)
[median (range)]	39 (19–77)
ART_3 _Treatment Experience [n (%)]	
Naive	168 (60)
Experienced	110 (40)
Baseline absolute CD4	
[mean (sd)]	201 (163)
[median (range)]	173 (0–883)
Baseline log_10 _HIV-1 Plasma Viral Load	
[mean (sd)]	4.9 (0.7)
[median (range)]	5.0 (2.7–6.3)
Days on new regimen	
[median (range)]	286 (30–1221)
Year of study entry [n (%)]	
2003	51 (18)
2004	103 (37)
2005	81 (29)
2006	43 (16)
New Regimen Type_4 _[n(%)]	
NNRTI & ≥ 2 NRTIs	63 (23)
PI_b _& ≥ 2 NRTIs	204 (73)
NNRTI & PI_b _& ≥ 1 NRTI	8 (3)
≥ 2 NRTI	3 (1)
# Adherence Scores per patient	
[median (range)]	4 (1–27)

1. MSM: men having sex with men.

2. IDU: injection drug use.

3. ART: antiretroviral therapy

4. NNRTI: non-nucleoside reverse transcriptase inhibitor; NRTI: nucleoside/nucleotide reverse transcriptase inhibitor; PI_b_: ritonavir boosted protease inhibitor.

Of the 1155 records in the final analysis dataset representing the longitudinal histories of 278 patients, HIV viral load and adherence were measured on the same date in 556 (48%) records. Of the 1155 records, 599 (52%) represented missing adherence scores at dates of viral load measurement. Of the 599 missing adherence scores, 426 were imputed using the last observation carried forward approach (LOCF) and 173 were imputed by backfilling values. Even though these missing adherence scores technically represent missing values at the time the viral load measures were taken, they conceptually represent values that were obtained at a different time point than the viral load measures. These instances typically represent patients for whom blood is drawn either before of after a clinic visit at which adherence assessment was conducted. The median (IQR) time between the regimen start date and date of the first recorded adherence score was 21 (13–60) days.

### Time to First Viral Suppression Analysis

**Figure 2:**
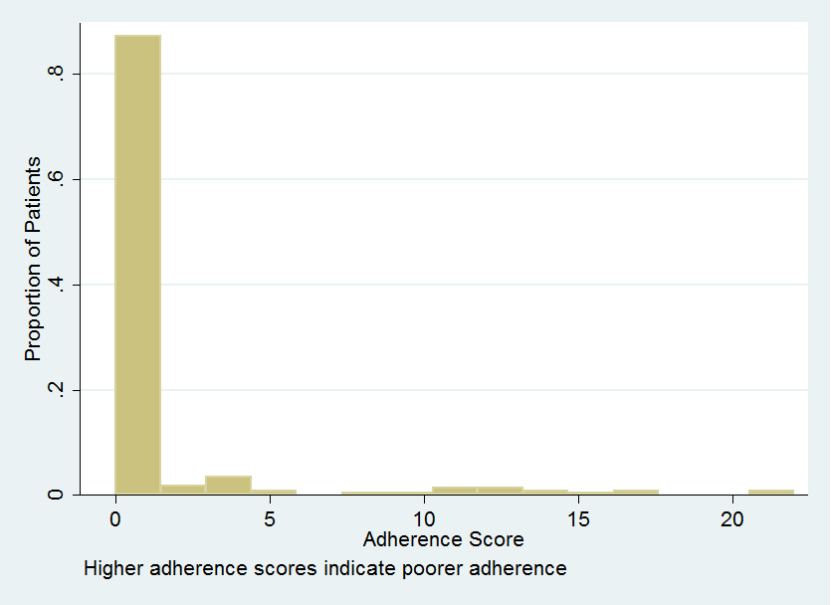
Distribution of first adherence scores during the study period (n = 278 patients).

**Figure 3:**
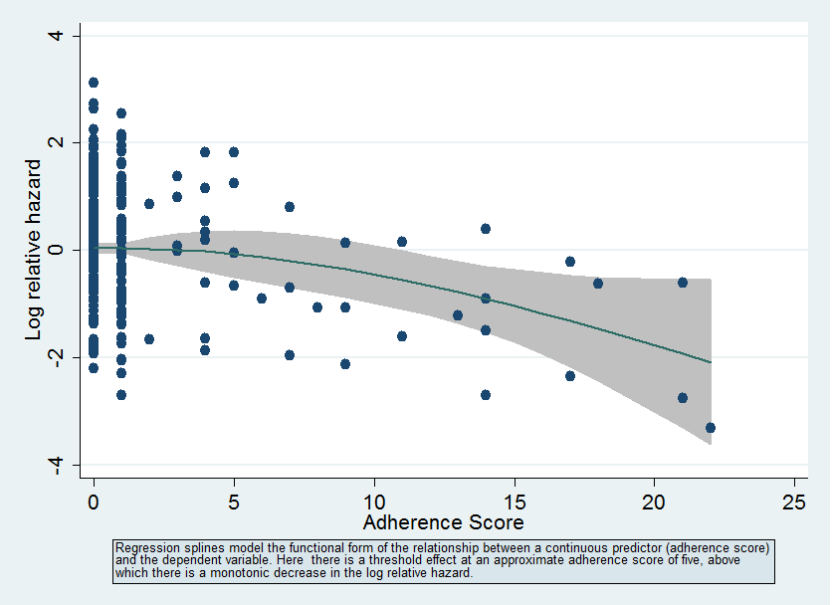
Regression spline (95% confidence interval) of adherence score in Cox model of time to viral suppression, adjusted for treatment experience and baseline viral load.

Because the distribution of adherence scores was highly skewed (Figure 2) we modeled adherence scores using binary indicator variables. In addition to adherence categories, the following potential covariates were examined in separate unadjusted Cox regression models: sex, race/ethnicity, HIV transmission risk factor, age, baseline CD4+ lymphocyte category (0–49, 50–199, ≥ 200), baseline log_10 _HIV plasma viral load, prior antiretroviral treatment experience (naïve, experienced), index regimen type. Of these potential covariates, baseline HIV viral load and race were significantly (p < 0.05) associated with time to viral suppression. Table 2 presents unadjusted and adjusted analyses of the effect of time updated adherence scores on time to viral suppression. Adjusted hazard ratios (HR) less than 1 are interpretable as indicating longer time to achieving viral suppression relative to the reference category. As anticipated, treatment experienced patients and those with higher baseline viral loads had longer times until achieving viral suppression. Race was not independently associated with the outcome in a model controlling for these two factors and adherence, and was therefore omitted from the final model. Controlling for the remaining two covariates (prior treatment status and baseline HIV viral load), having a time-updated adherence score of five or more (the lowest detectable cut point after Bonferroni correction of overall Type I error rate) was significantly predictive of longer time to achieve viral suppression. There were no 2-way statistical interactions (p > 0.10) between adherence score and either baseline viral load or prior treatment experience. The functional relationship between covariate-adjusted adherence sum score modeled as a regression spline and the log (HR)+residual is presented in Figure 3.

**Table 2:** Unadjusted and adjusted effects of time-updated adherence scores on time to first HIV viral load ≤ 400 copies/ml in Cox regression models (n = 278 patients)

	Unadjusted			Adjusted		
Predictor	HR_1_	95% CI	p-value	HR	95% CI	p-value

Adherence Score						
< 5	1.0			1.0		
≥ 5	0.42	0.22–0.79	0.007	0.36	0.19–0.69	0.002
Antiretroviral Experience						
Naïve	1.0			1.0		
Experienced	0.79	0.60–0.1.05	0.10	0.68	0.50–0.91	0.01
Baseline log_10 _HIV viral load						
	0.82	0.68–0.99	0.04	0.71	0.58–0.87	0.001
Race			0.047			
White	1.0					
Black	1.42	0.92–2.19	0.11	---	---	---
Hispanic	1.51	1.10–2.06	0.01			
Unknown/Other	1.01	0.63–1.62	0.98			

HR: hazard ratio

### Maintenance of Viral Suppression Analysis

**Figure 4:**
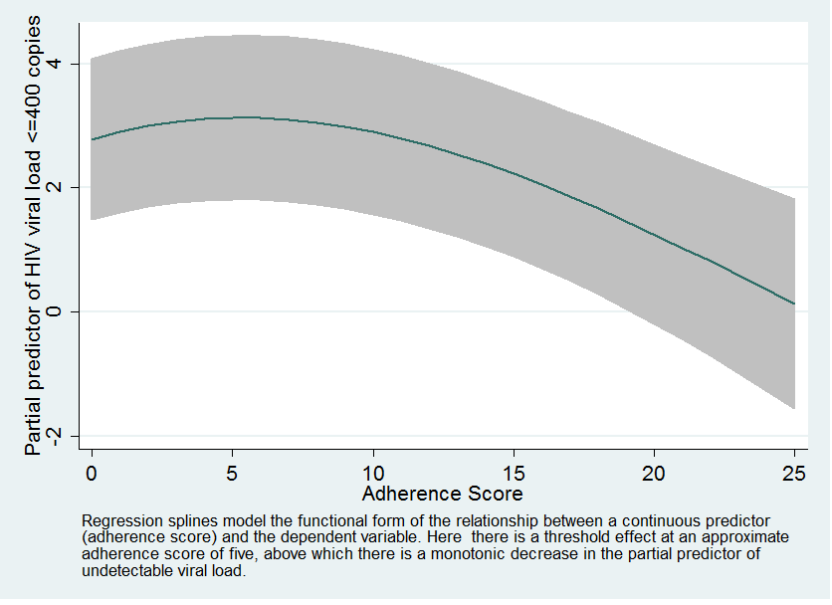
Regression spline (95% confidence interval) of adherence score in GEE logit model of maintained viral suppression, adjusted for treatment experience and baseline viral load.

Table 3 presents the results of unadjusted and adjusted effects of time-updated adherence scores on maintenance of viral suppression in population averaged GEE logit regression models. The table reports crude and adjusted odds ratios of final models. The same potential covariates were examined as those reported above for the time to initial suppression analysis. With the exception of the time-updated adherence scores, none of the examined covariates were significantly associated with maintenance of viral suppression in unadjusted analysis. Prior treatment experience and baseline plasma viral load were included in the adjusted model to maintain comparability with the time to initial viral suppression analysis (Table 2). In both unadjusted and adjusted models, the lowest detectable cut point on adherence score was the same as that observed in the time to initial viral suppression analysis (≥ 5/< 5). The functional relationship between covariate-adjusted adherence sum score modeled as a regression spline and the partial predictor of viral suppression is presented in Figure 4.

**Table 3:** Unadjusted and adjusted effects of time-updated adherence scores on maintenance of HIV viral load ≤ 400 copies/ml in generalized estimating equation logit regression models (n = 217 patients achieving initial viral suppression)

	Unadjusted			Adjusted		
Predictor	OR_1_	95% CI	p-value	OR	95% CI	p-value

Adherence Score						
< 5	1.0			1.0		
≥ 5	0.20	0.05–0.79	0.02	0.17	0.05–0.66	0.01
Antiretroviral Experience						
Naïve	1.0			1.0		
Experienced	0.78	0.28–2.24	0.65	0.60	0.21–1.70	0.34
Baseline log_10 _HIV viral load						
	0.56	0.26–1.23	0.15	0.49	0.22–1.11	0.09

OR: odds ratio

## Discussion

In the developmental phase of adherence measurement in our clinic, we constructed a 5-item instrument whose individual items were selected from the 51-item ACTG adherence battery [2] on the basis of factor structure and internal consistency reliability. In the manuscript presenting this developmental work, we showed that responses on the 5-item adherence index, administered on one occasion 30 days after initiating a new antiretroviral regimen, were moderately correlated (Spearman rho 0.40 – 0.48) with measures of electronic drug monitoring (EDM) and were predictive of HIV viral load responses at 3 and 6 months after start of treatment in models controlling for baseline viral load and prior antiretroviral experience. We also showed that a cut point of 5 or more on the index distinguished those with viral load suppression (≤ 400 copies/ml) at 3 and 6 months from those failing to suppress at the same time points [1]. The currently reported analyses were conducted to evaluate whether the same 5-item index, when administered repetitively under longitudinal follow up, predicted initial viral suppression and maintenance of suppression while patients continued the index regimen. We found, conditional upon the study eligibility criteria and analytic methods, that the self-report adherence index scores were predictive of both outcomes in models controlling for prior antiretroviral treatment experience and baseline plasma viral load. For the time to initial viral suppression outcome, adherence scores ≥ 5 were associated with an approximately 60% reduced hazard of achieving a plasma viral load ≤ 400 copies/ml. For the maintenance of viral suppression outcome, adherence scores ≥ 5 predicted an approximately 80% lower chance of maintaining viral suppression relative to scores less than 5.

These findings are not directly comparable to the effects demonstrated in our earlier study for several reasons including: (1) period effects (1998 – 1999 vs. 2003 – 2006) associated with changes in potency and simplicity of antiretroviral regimens; (2) differences in prior treatment experience (22% vs. 60% antiretroviral naïve comparing the earlier to the current study); (3) conditions of adherence measurement (written completion [earlier study] vs. computer assisted [current study]); and (4) differences in analytic approach (outcomes analyzed cross sectionally at fixed time points [earlier study] vs. longitudinally in continuous time [current study]). Nonetheless, the current results contribute to the predictive validation of the instrument as it has been used in routine clinical care of patients on antiretroviral therapy.

In a recent review of the status of HIV adherence measurement, Chesney presented a conceptual model of adherence assessment and intervention, distinguishing research from clinical applications, and resource-rich from resource-poor settings. In discussing the "elusive gold standard" of adherence measurement, she emphasized that "efforts should continue to develop a portfolio of different valid and reliable self-report measures with varying strengths and weaknesses that can be optimally applied, depending on the situation [3]." In that spirit, we discuss a number of challenges that emerged in exploring the relationship between routine longitudinal adherence measurement using the Owen Clinic instrument and viral suppression.

**Figure 1:**
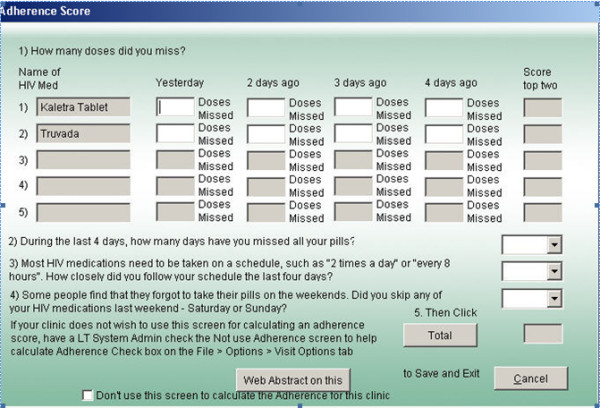
Screen shot of adherence instrument.

First, adherence score distributions in the current (Figure 2) and previous study were highly skewed, with most observations clustered in a range reflecting good adherence and the remainder of observations distributed in the long tail of the distribution reflecting poorer adherence. The clustering of observations toward the excellent adherence end of the distribution creates *ceiling effects *[4]. Others have noted the same phenomenon for other self report measures [5-8]. The clustering of scores toward excellent adherence likely represents a mixture of responses from truly adherent patients and from others exhibiting *social desirability bias *[9]. Simoni et al have commented on approaches to minimize both ceiling effects and social desirability bias in adherence assessment [10]. Comparison of self report scores to independent and hopefully more objective measures of adherence (e.g. pharmacy refill data, pill counts, EDM) offer an opportunity to assess the effect of social desirability bias. In other contexts, the use of measures designed to measure social desirability as a construct have been used as covariates to explain self reported health behaviors subject to such response bias [11,12]. With regard to ceiling effects not contaminated by social desirability bias, designing items to capture more challenging aspects of adherence behavior, such as timing of doses or dose taking at inconvenient times (e.g. at work, on weekends, or in the presence of persons not knowing the patient's diagnosis), has been recommended to mitigate the strict ceiling commonly observed in self reported adherence. It should be noted, however, that our instrument included three items (Figure 1: items 2–4) dealing with such recommended approaches.

Second, the modeling of adherence score is not straightforward. As constructed its scale of measurement is discrete numerical with a possible range of 0 – 25 with skewness not amenable to a normalizing transformation. Although cut point selection for an underlying numerical measure may introduce bias in effect measurement [13] and may reduce power to detect effects in comparison with use of the numerical measure [14], cut point models are often preferred because of simplicity of data summarization and interpretation. Post hoc cut point selection, as pointed out by the authors of the STARD initiative [15] (Item 9), may not be replicable with other datasets. In our modeling of the effect of adherence score, we employed an approach adapted from Williams et al [16], first exploring the functional form of the relationship between adherence score as a numerical measure using smoothing regression splines as implemented by Royston and Sauerbrei in STATA followed by cut point examination adjusted for multiple comparisons [17]. Cut points alternative to what we have described as the lowest detectable cut points could be recommended if alternate methodologies of correction for multiple comparisons were employed (e.g. cross validation or split sample approaches, or examination in independent data sets). It is of interest that in our earlier study, a similar cut point on the same instrument (≥ 5/< 5) was felt to be the most discriminating cut point [1]. After examining the regression spline plots for both outcome metrics (Figures 3 and 4) in the current study, we felt that a cut point around 5 identified a region above which a monotonic relationship between adherence score and functions of the outcome metrics was suggested. In clinical care settings, we believe, based on these data, that our clinicians should be alert to clinically significant problems with adherence for scores at or above 5.

Third, because of the observational nature of the data, measurements of adherence and HIV plasma viral load were not scheduled to occur simultaneously. Typically clinicians order viral loads every 3 – 6 months depending on clinical factors. Adherence in contrast is measured in our clinic at all routine visits. Conceptually, adherence is a construct representing a daily health behavior for which various self-report indicators have been developed and mapped to estimates of percentage adherence over a defined period or, as in the case of the Owen Clinic instrument, given interpretability primarily through demonstrated association with viral suppression. Because of the staggered nature of data accrual in the clinic, decisions must be made regarding how to line up sequential viral load and adherence measures. At a conceptual level, it is a non-trivial question to decide over how long a period an adherence measure based on a limited recall period (4 days in the case of our instrument) can be extrapolated with regard to preceding and future adherence behaviors for which the self-report data represents an imperfect indicator. In our primary analysis, we made the assumption that a given adherence assessment carried forward no longer than 90 days from the antecedent adherence measurement. Whether the observations that are not temporally matched represent truly missing observations is debatable since the very nature of the data accrual process in clinical care did not require temporal matching of adherence and viral load measurement. Because the LOCF principle has been criticized in recent years [18], we explored alternate analyses to evaluate the robustness of our findings. First, to determine if the frequency of adherence measurement was related to adherence scores such that longer intervals between measurements were associated with better or poorer adherence, we calculated rates of adherence measurement per 100 days of follow up. We then divided the adherence measurement rate distribution into quartiles and used analysis of variance to test for equality of mean adherence scores across the quartiles, finding no significant difference (p = 0.89). This provided limited evidence that, in our data set, adherence scores were not systematically related to frequency of measurement, although others have found that missing adherence values were associated with nonadherence [19]. Second, we restructured the data set by grouping follow up time in 6 month intervals, taking the median adherence score for the interval as representative, the last viral load in the interval as the outcome, and repeating the panel regression for longitudinal viral suppression. In a model comparable to that shown in Table 3 controlling for prior treatment experience and baseline log_10_-HIV viral load, the adjusted odds ratio for viral suppression was 0.14 (95% CI: 0.06 – 0.33, p < 0.0001) for a 6-month median adherence score greater than 5. Finally, in a third analysis of maintenance of longitudinal viral suppression, mean adherence scores were calculated for the period immediately prior to each viral load measurement, creating a score for each interval between viral load measurements. This operationalization of adherence was then fit in a GEE logit model for maintenance of viral suppression, again controlling for prior treatment experience and baseline log_10_-HIV viral load. The adjusted adherence odds ratio for maintaining viral suppression for a mean interval adherence score greater than 5 was 0.28 (95% CI: 0.14 – 0.57, p < 0.0001) Therefore, although the adherence effect estimates were model dependent, the direction of effect was consistent and significant across models.

## Conclusion

Despite the limitations of self-report adherence measures, they are likely to remain the most frequent modality of adherence assessment in clinical settings. The brief self-report instrument examined in this study and in an earlier developmental study has been demonstrated to correlate with electronic drug monitoring and to be predictive of viral load responses both when administered at baseline and also when administered in longitudinal follow up of unselected patients in clinical care for HIV infection.

## Methods

A retrospective observational cohort study was conducted including all HIV-infected adults under care at the UCSD Owen Clinic between January 2003 and June 2006. Patients were included in the analyses reported here if they: (1) had at least one self report medication adherence score recorded; (2) either initiated antiretroviral therapy for the first time or began a new regimen during the study period; (3) had a plasma viral load ≥ 400 copies/ml prior to initiation of the index regimen; (4) had at least one post baseline plasma viral load; and (5) remained on the index regimen for at least 30 days. Only time on the first regimen during the study period (index regimen) is included in reported analyses. During the study period, patients on antiretroviral therapy were asked to complete, prior to meeting with their medical provider, a computer-assisted four item antiretroviral medication adherence survey [20] (Figure 1) at every primary care visit. The adherence assessment takes 2–3 minutes to complete and is overseen by the medical assistant who is also recording vital signs. Clinicians review adherence scores and are expected to document adherence counseling in the clinic electronic medical record if scores indicate adherence problems. The adherence items are a subset of the AIDS Clinical Trials Group (ACTG) adherence battery [2]. Items 1 and 2 query the number of missed doses of each antiretroviral medication over each of the preceding four days. The number of missed doses for each drug is summed across all four antecedent days. The sum scores of the two drugs with the highest number of missed doses (designated items 1 and 2) are included in the index score. Item 3 asks "During the past 4 days, on how many days have you missed all your pills?" (response options (numeric code): no days (0), one day (1), two days (2), three days (3), all four days (4)). Item 4 inquires "How closely did you follow your specific schedule over the last 4 days?" (response options (numeric code): never (4), some of the time (3), about half the time (2), most of the time (1), all of the time (0)). Item 5 deals with weekend adherence behavior asking "Did you skip any of the HIV medications last weekend – last Saturday or Sunday?" (response options (numeric code): no (0), yes (1)). The index score is the sum of responses to the four items with a possible range of 0 (best adherence) to 25 (poorest adherence) if each component of the regimen was dosed twice daily.

Two outcome measures were operationally defined as: (1) time to first virologic suppression defined as HIV plasma viral load (pVL) ≤ 400 copies/ml after regimen initiation; and (2) maintenance of virologic suppression (pVL ≤ 400 copies/ml). Follow up time for each patient began with the date of initiation of the index antiretroviral regimen and ended with the earliest of the following events: (1) change or discontinuation of the index regimen; (2) last clinic visit date; or (3) end of the study period. Time to first virologic suppression on the index regimen was examined using extended Cox models incorporating time-updated adherence scores. It was confirmed that the proportional hazards assumption was met for all covariates included in the Cox models using log(t) by covariates interactions [21]. Maintenance of virologic suppression was evaluated in logit models using population-averaged generalized estimating equations (GEE) with time varying covariates [22,23]. GEE are a family of methods suitable for the analysis of the longitudinal relationship between a continuous or dichotomous outcome variable and both time-dependent and time independent covariates. The within subject dependency of observations is handled by assuming a working correlation structure for the repeated measurements of the outcome variable [24]. The analysis for the maintenance of virologic suppression analysis included only those patients who achieved an initial pVL ≤ 400 copies/ml and their follow up began on the date of initial virologic suppression. The primary independent variable was time-updated adherence score. Because adherence scores were highly skewed toward higher scores (reflecting poorer adherence), adherence scores were first fit using univariate regression splines to examine the functional relationship between adherence score and the outcome measures [16,17]. Spline techniques are a family of methods for determining the functional form of the relationship between a continuous predictor variable (e.g. adherence score) and an outcome variable [25]. After determining that the functional relationships were approximately monotonic, eight binary cut points on adherence score were examined in ascending order (e.g. ≥ 1/< 1, ≥ 2/< 2, ≥ 3/< 3) until a threshold demonstrating statistical significance in adjusted models was found (lowest detectable cut point). Because multiple ascending potential cut points were examined, tests of significance for adherence score were adjusted using the Bonferroni method to maintain a overall type I error rate of 0.05 [16,26]. Thus the critical p-value for each cut point was 0.05/8 = 0.00625. Examined covariates included: age, sex, race/ethnicity, HIV transmission risk factor, treatment experience (naïve or experienced at time of index regimen initiation), regimen type (number and type of antiretroviral drug classes in the regimen), and both CD4 and pVL measured at the closest time prior to initiation of the index regimen.

Because HIV plasma viral load and adherence score were not always measured on the same dates, records with missing values for adherence score after the first adherence measurement date were imputed using the last observation carried forward (LOCF) principle. Because the first adherence measurement date usually occurred after the regimen start date, records with missing early adherence scores were backfilled to the regimen start date using the score of the first adherence measurement. Adherence scores were carried forward and backfilled no more than 90 days from the temporally closest adherence measurement date. Viral load data were not carried forward.

Statistical analyses were performed using Stata 10.0 (Stata Corporation, College Station, TX). This research was approved by the University of California San Diego Human Subjects Committee (Project No. 040394)

## Competing interests

The authors declare that they have no competing interests.

## Authors' contributions

WCM designed the study, conducted the final analysis, and prepared the manuscript; EB and EW conducted extensive medical record review and prepared preliminary analysis of the data; CB and BC contributed to design of the study and manuscript preparation; SM advised on statistical analysis and contributed to the manuscript preparation. All authors read and approved the final manuscript.
